# Occurrence of *Trichinella* Species in Wild Boar (*Sus scrofa*) Populations Across Bulgaria

**DOI:** 10.3390/ani16040648

**Published:** 2026-02-18

**Authors:** Nikolay Lalkovski, Francesco Celani, Daniele Tonanzi, Gianluca Marucci

**Affiliations:** 1 National Diagnostic and Research Veterinary Institute, 1606 Sofia, Bulgaria; laleto72@abv.bg; 2 Unit of Foodborne and Neglected Parasitic Diseases, Department of Infectious Diseases, Istituto Superiore di Sanità, Viale Regina Elena 299, 00161 Rome, Italy; francesco.celani@iss.it (F.C.); daniele.tonanzi@iss.it (D.T.); 3 European Union Reference Laboratory for Parasites, Department of Infectious Diseases, Istituto Superiore di Sanità, Viale Regina Elena 299, 00161 Rome, Italy

**Keywords:** *Trichinella britovi*, wild boar (*Sus scrofa*), Trichinellosis, Bulgaria

## Abstract

Trichinellosis is a natural-focal helminthic zoonosis that affects the skeletal muscle tissue of carnivorous and omnivorous wild and domestic animals. In Europe, four prevalent species belonging to the genus *Trichinella* have been identified: *T. spiralis*, *T. britovi*, *T. nativa* and *T. pseudospiralis*. The aim of this study was to identify the *Trichinella* species circulating in wild boar populations in Bulgaria, and to evaluate their prevalence. The research covered five hunting seasons, from October 2020 to February 2025. A total of 43,228 wild boar carcasses were examined using artificial digestion, and 597 tested positive, corresponding to a prevalence of 1.5%. The *Trichinella* isolates were identified at the species level by multiplex PCR at the European Union Reference Laboratory for Parasites in Rome, Italy. Among the samples suitable for molecular identification, *Trichinella britovi* was the only species detected in wild boar in Bulgaria during the study period, although other *Trichinella* species, including *T. spiralis* and *T. pseudospiralis*, have occasionally been reported in this host in earlier investigations. Geographical distribution analysis showed that most infected wild boar originated from southern part of the country.

## Simple Summary

Trichinellosis is a disease caused by parasitic worms that can be transmitted from animals to humans. Free-ranging wild boar play an important role in maintaining this parasite in nature and are also a potential source of human infection through the consumption of raw or undercooked meat. The aim of this study was to identify which species of this parasite are present in wild boar in Bulgaria and to determine the frequency of infection. The study was conducted over five hunting seasons, from 2020 to 2025, and more than 43,000 wild boar carcasses were examined. Approximately 1.5% of the animals were found to be infected. All positive samples belonged to a single parasite species, *Trichinella britovi*, even though other species have been occasionally reported in the past. Most infected wild boar originated from the southern part of the country. These findings provide valuable information for public health protection, food safety, and wildlife management by helping authorities better assess infection risk and design effective control measures.

## 1. Introduction

Trichinellosis is a foodborne zoonotic disease caused by species of the genus *Trichinella*, which includes ten recognized species and three additional genotypes. Four of these species, *T. spiralis*, *T. britovi*, *T. nativa*, and *T. pseudospiralis*, are prevalent in Europe [1]. In Bulgaria, trichinellosis represents one of the most important parasitic zoonoses.

Surveillance data indicate that the prevalence of *Trichinella* infection in wild boar in Bulgaria has historically been relatively high. According to the European Food Safety Authority (EFSA), prevalence declined from 1.25% in 2015 to 0.51% in 2018, with annual values of 1.25% (2015), 1.00% (2016), 0.95% (2017), and 0.51% (2018). During the same period, the incidence of confirmed human infections ranged from 0.31 to 0.77 per 100,000, whereas the average incidence in the European Union was considerably lower, ranging from 0.01 to 0.03 per 100,000.

Two species, *T. spiralis* and *T. britovi*, have been identified as the etiological agents of trichinellosis in Bulgaria [2,3]. The main source of human infection (58.6% of 29 outbreaks) was the consumption of raw or undercooked meat products, mainly sausages, prepared from wild boar meat [4]. The same *Trichinella* species have also been identified in wild boar, underscoring the importance of this host in the transmission of infection to humans. In samples examined between 2010 and 2016, the ratio of *T. britovi* to *T. spiralis* in wild boar populations was estimated at 45:1 [5].

The aim of this study was to describe the *Trichinella* species detected in wild boar in Bulgaria and their distribution across the country, based on surveillance carried out during five hunting seasons (2020–2025), thereby contributing to an improved understanding of species-specific prevalence in this host.

## 2. Materials and Methods

**Figure 1:**
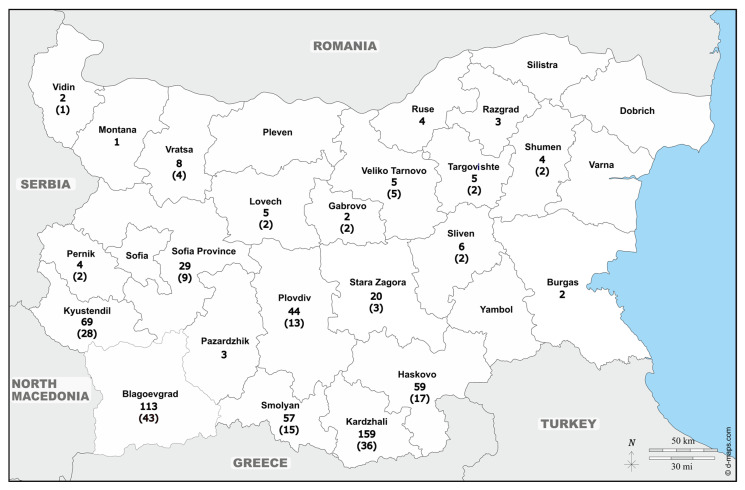
Geographical distribution of *Trichinella*-positive wild boar carcasses in Bulgaria in the 2020–2025 period. Values represent absolute counts of positive samples per region; *T. britovi* isolates are shown in brackets. The Black Sea is colored azure on the map.

The research was conducted between October 2020 and February 2025, covering five hunting seasons. In Bulgaria, wild boar hunting begins in October and continues until the second weekend of January. Muscle tissue samples were collected from wild boars shot during regular hunting activities in several regions of Bulgaria (Figure 1). Ten grams of tissue were sampled from diaphragm of each animal and tested by artificial digestion according to ISO18743:2015 [6] at regional laboratories in charge for official controls. *Trichinella* larvae originating from positive animals were collected, stored in 90% ethanol, and sent to the European Union Reference Laboratory for Parasites (EURL-P; https://www.iss.it/en/eurlp-chi-siamo) (Rome, Italy) for species identification by multiplex PCR [7]. Briefly, DNA was purified from single larvae using a DNA IQ System kit (Promega, Madison, WI, USA) and a Tissue and Hair Extraction kit (Promega, USA). Five primer sets, targeting specific regions of the ribosomal DNA repeats (expansion segment V, ITS1 and ITS2), were used in multiplex PCR to obtain a species-specific electrophoretic DNA banding patterns [8,9].

## 3. Results

A total of 43,228 wild boar carcasses were examined using artificial digestion, of which 597 tested positive for *Trichinella*, corresponding to a prevalence of 1.5% (Figure 1, Table 1). Numerous larvae were visually observed in all positive samples; however, infection intensity could not be quantified due to the absence of larvae per gram measurements. Out of a total of 597 positive isolates, a subsample of 238 was submitted to the EURLP for species identification via multiplex PCR. The subsample included only the larvae which, under microscopic observation, showed to be in acceptable condition for analysis (i.e., intact cuticle and visible internal structures). Specifically, these included 23 isolates from animals hunted during the 2020–2021 season, 68 from 2021 to 2022, 39 from 2022 to 2023, 65 from 2023 to 2024, and 43 from 2024 to 2025. Most isolates (68%) submitted to EURLP for species identification originated from the southern part of the country. PCR products specific for *Trichinella britovi* were obtained from 186 samples (78.2%) of the 238 samples. For the remaining 52 samples, species identification was unsuccessful, likely due to insufficient DNA recovery resulting from sample degradation during storage.

## 4. Discussion

In Europe, expanding wild boar populations pose a potential risk for the spread of *Trichinella* spp. due to their high susceptibility to this nematode [10]. Wild mammals are common reservoir hosts, and parasite biomass is higher in wild animals than in domestic ones [11]. Although most trichinellosis outbreaks in Eastern Europe remain linked to pork consumption, especially from pigs raised under uncontrolled housing conditions [12,13], cases associated with game meat, particularly wild boar, are increasing. In some countries, such as Croatia and Poland, wild boar meat has even surpassed pork as the main source of human infection [14,15,16]. Despite this, the prevalence of *Trichinella* in European wild boar populations has remained relatively stable in recent years [17], while the number of wild boars continues to rise [18,19,20]. For instance, in Bulgaria, according to data provided by the Executive Forest Agency, the number of wild boars recorded in spring 2025 was 64,840, representing a 10.2% increase compared to 2024 [21].

Bulgaria is geographically divided by the Balkan Mountains into northern and southern regions. The north comprises the Danubian Plain and low hills, with a continental climate of cold winters and hot summers, and vegetation dominated by grasslands, agricultural fields, and mixed forests. Southern Bulgaria is more mountainous, featuring ranges such as the Balkan, Rhodope, Rila, and Pirin, with denser forest, richer undergrowth, and varied landscape. Most wild boars inhabit the south, at an approximate 2:1 ratio compared to the north. Habitat studies indicates that wild boars occupy about 57.5% of the national territory, favoring broad-leaved and continuous forests, particularly in the southern and southwestern regions, whereas northern areas are less suitable due to the extensive agricultural land [22].

Recent changes in wild boar population size may interact with other ecological factors affecting *Trichinella* circulation. For example, in Germany, an increase in *Trichinella* prevalence in wild boar populations between 2013 and 2023 occurred in areas inhabited by predator species such as raccoon dogs and wolves [23]. Similarly, in Poland, *Trichinella* infection in wild boars correlated positively with raccoon dog density, suggesting that these carnivores help maintain the sylvatic cycle. [24]. These findings indicate that parasite prevalence is influenced not only by host population size but also by the abundance and distribution of other competent hosts. Accordingly, increases in wild boar numbers, such as those observed in Bulgaria, have been hypothesized to interact with the presence of other reservoir species, potentially affecting *Trichinella* transmission dynamics. In this context, the raccoon dog, present in Bulgaria since 1968, is now widely distributed across the country. There are 75 recorded sightings covering almost the entire territory, with the majority (77.4%) occurring at altitudes between sea level and 199 m a.s.l., and 56% located within national protected areas [25]. However, no data on *Trichinella* infection in raccoon dogs were collected in this study. Thus, their presence is discussed here only as a potential ecological factor based on existing literature, rather than as evidence of causal role in the sylvatic cycle. By interacting with local wild boar populations, raccoon dogs could potentially contribute to the maintenance and spread of the parasite.

Epidemiological data from October 2020 to February 2025 indicate higher *Trichinella* prevalence in southern Bulgaria, with 558 out of 597 positive cases reported there, compared to only 39 cases from the northern regions. Although these data align with the previously discussed ecological patterns, this study did not directly assess the relationship between wildlife population dynamics, the presence of other reservoir hosts, and *Trichinella* prevalence.

The detection of *T. britovi* in the tested animals suggests its predominance in Bulgarian wild boar and is consistent with historical data. However, species identification was performed on a subset of positive samples only, and therefore the presence of other *Trichinella* species among the non-typed isolates cannot be excluded. Between 2000 and 2024, a total of 261 wild boar tested positive for *Trichinella*: 253 (97%) were infected with *T. britovi*, seven (2.7%) with *T. spiralis*, and one (0.3%) with *T. pseudospiralis* [26]. Taken together, these data support the view that *T. britovi* is the principal *Trichinella* species circulating among wild mammal populations in Bulgaria.

## 5. Conclusions

The increasing wild boar population in Bulgaria, particularly in the forested southern regions, may influence *Trichinella* transmission in wildlife. However, parasite prevalence may be influenced not only by wild boar abundance but also by the presence of other competent hosts, such as the raccoon dog, which is now widely established across the country. In this study, *T. britovi* was the only species detected among the analyzed isolates, consistent with historical records that suggest its dominance in Bulgarian wild boars, although the presence of other *Trichinella* species cannot be excluded. An effective management of zoonotic risk should therefore combine the monitoring of wild boar populations with the assessment of other wildlife hosts, and the evaluation of habitat conditions to prevent trichinellosis linked to the consumption of game meat.

## Figures and Tables

**Table 1:** Number and prevalence of *Trichinella britovi* positive samples detected per calendar year (October 2020–February 2025).

Year	No. of Tested Animals	No. of Positive Animals	Prevalence (%)
2020	6857	59	0.86
2021	8665	141	1.63
2022	7657	115	1.5
2023	8650	122	1.41
2024	9968	127	1.27
2025	1431	33	2.3
Overall	43,228	597	1.5

## Data Availability

The datasets generated and analyzed during the current study are publicly available in the Database of the International Trichinella Reference Center at [https://trichinella.iss.it/Database.aspx].
